# Case report: a rare case of attempted homicide with *Gloriosa superba* seeds

**DOI:** 10.1186/s40360-016-0069-6

**Published:** 2016-06-21

**Authors:** Chaminda J. Kande Vidanalage, Rohan Ekanayeka, Deepthi K. Wijewardane

**Affiliations:** General Hospital Chilaw, Putlam Road, Chilaw, Sri Lanka; 259/2d Kahanthota, Malabe, Colombo, Sri Lanka

**Keywords:** *Gloriosa superba* seeds, Poisoning, Colchicine, Sri Lanka

## Abstract

**Background:**

*Gloriosa superba*, well known as the glory lily or superb lily, is a tropical climbing plant that features an exotic red flower. The plant is poisonous because of high concentrations of colchicine in all parts of the plant. It is commercially grown for use in Ayurveda medicine and as a cash crop for extracting colchicine in India and Africa. It is a wild plant in Sri Lanka and commercial cultivation is rare. Accidental and suicidal poisonings with *Gloriosa* tubers are well known and reported. There are no case reports of poisoning by *Gloriosa* seeds in Sri Lanka. Google and PubMed searches showed no reported cases of poisoning with seeds or their use with homicidal intent in other parts of the world.

**Case presentation:**

A 27-year-old man was brought to hospital with profuse vomiting and diarrhea after drinking coriander tea, which is a common traditional treatment for common cold. The family members suspected poisoning by *Gloriosa* because they had seeds at home and the victim’s sister-in-law who had made the herbal tea went missing from home. They were able to identify *Gloriosa* seeds, which looked similar to coriander, in the pot. The patient developed shock and respiratory distress and needed ventilation and intensive care. He also developed mild renal impairment, and thrombocytopenia. He developed massive generalized alopecia while recovering from acute illness. Full recovery was achieved after 15 days of hospital care.

**Conclusions:**

There are many poisonous plants in Asian countries. This case highlights the possibility of accidental or intentional use of *Gloriosa* seeds or its extracts to cause potentially fatal poisoning. It would be difficult to identify *Gloriosa* as the cause of poisoning without any background information because of multiple complications that can mimic a systemic infection. This case is a good example of the use of plants as biological weapons.

## Background

*Gloriosa superba* is a plant in the family Colchicaceae. It grows in the tropical climates of Africa, in Asian countries, including India, Sri Lanka, Malaysia, and Burma, and in Australia and Pacific islands. It is a branching climber that grows to about 5 m. The most common English names for the plant are flame lily, glory lily, superb lily, and creeping lily, which refer to its exotic flower. In Sri Lanka, it called *Niyagala* in Sinhalese, and in India it is called *Karthigaipoo* in Tamil language. It is the state flower of Tamil Nadu and is also the national flower of Zimbabwe. In Tamil Nadu, *Gloriosa* cultivation is promoted by government subsidy schemes and several hundred acres are grown as a cash crop. All parts of the plant are poisonous because of the high content of colchicine, which is a medicinal alkaloid, and the seeds are used to extract colchicine. Accidental poisoning and suicidal misuse of tubers are well known in areas where the plant grows [1]. In addition to colchicine, the plant also contains other compounds such as 3-desmethyl colchicine, beta-lumicolchicine, *N*-formyldesacetyl colchicine, 2-desmethyl colchicine, chelidonic acid, and salicylic acid [2]. The lethal dose of colchicine in humans is about 0.8 mg/kg [3, 4]. Generally, the colchicine content is higher in cultivated plants than in naturally grown plants, and the seed contains more colchicine than the tubers [5]. Colchicine can be extracted from the plant parts in boiling water [6], and extracting the seed can give a high concentration of colchicine in the boiled water. Colchicine is rapidly absorbed from the intestine and undergoes significant first-pass hepatic metabolism. The metabolites undergo enterohepatic circulation and are subsequently excreted in feces, leading to extended exposure of the intestine to the toxic effects. Renal clearance accounts for about 10–20 % of colchicine excretion [7].

Colchicine inhibits the polymerization of microtubules and formation of mitotic spindle in cell division. Therefore, the rapidly dividing cells of the intestinal mucosa are severely affected. Colchicine can cause severe gastroenteritis, shock, multiple organ failure, electrolyte imbalance, metabolic acidosis, pancytopenia, hypotension, rhabdomyolysis, hypocalcemia, and adult respiratory distress syndrome (ARDS) [8]. In addition, it can cause respiratory failure because of paralysis of intercostal muscles caused by ascending polyneuropathy [9]. Cases of toxic encephalopathy and progressive paralysis of the central nervous system and peripheral nervous system have also been reported [10], and cardiotoxicity with ST elevation on ECG, renal impairment, and thrombocytopenia are known [11]. Generalized massive alopecia after the acute stage of poisoning is also a well-recognized complication [12, 13]. Accidental or suicidal poisoning by *Gloriosa* tubers is well known in Sri Lanka. A hospital-based study in western Sri Lanka showed that out of 4556 cases of poisoning, 2.5 % were caused by plants and mushrooms, and *Gloriosa superba* was the commonest plant poison, being responsible for 44 % of them [14]. However, a literature search did not find any Sri Lankan cases of seed poisoning. Use of seed as a homicidal poison has never been described in Sri Lanka and PubMed and Google searches of “Gloriosa and seed” poisoning did not reveal any case reports.

## Case presentation

**Fig. 1:**
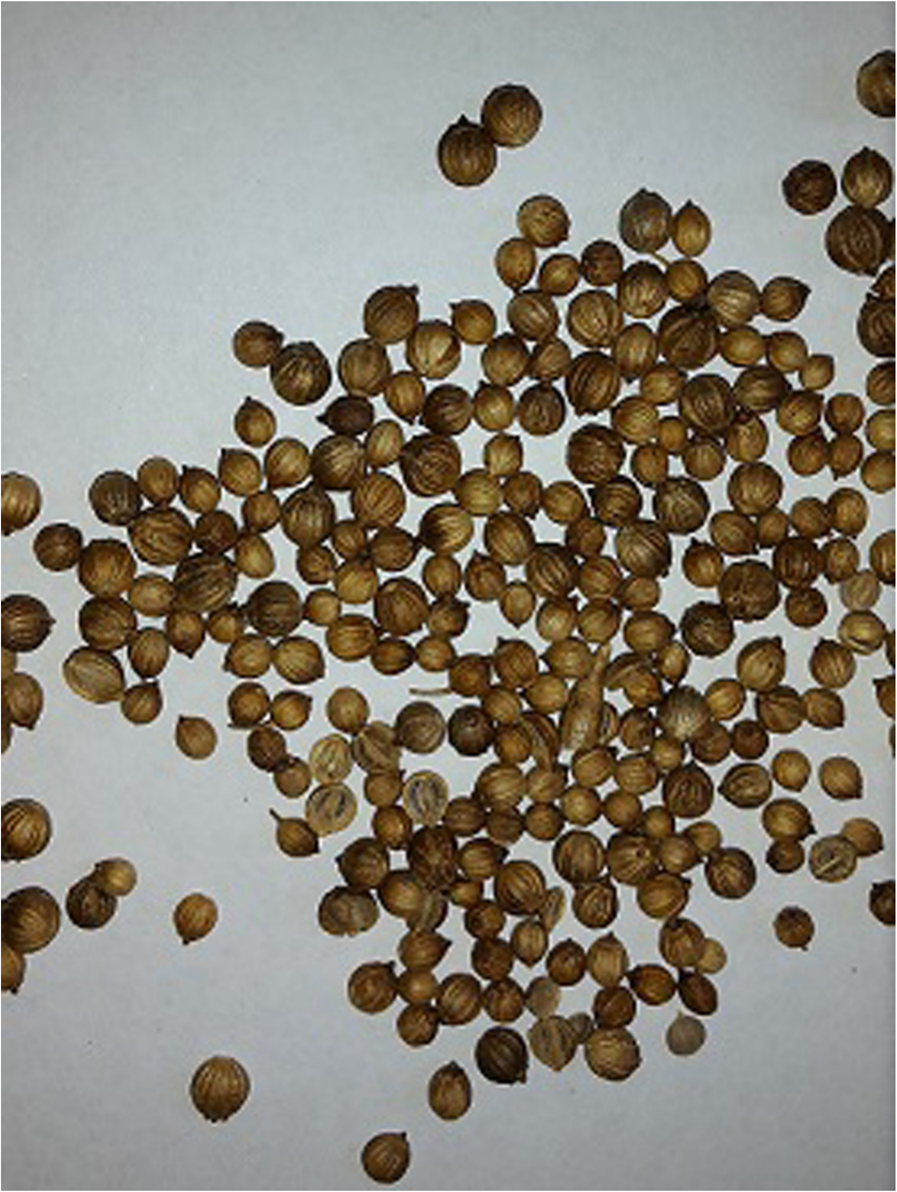
Coriander seeds

**Fig. 2:**
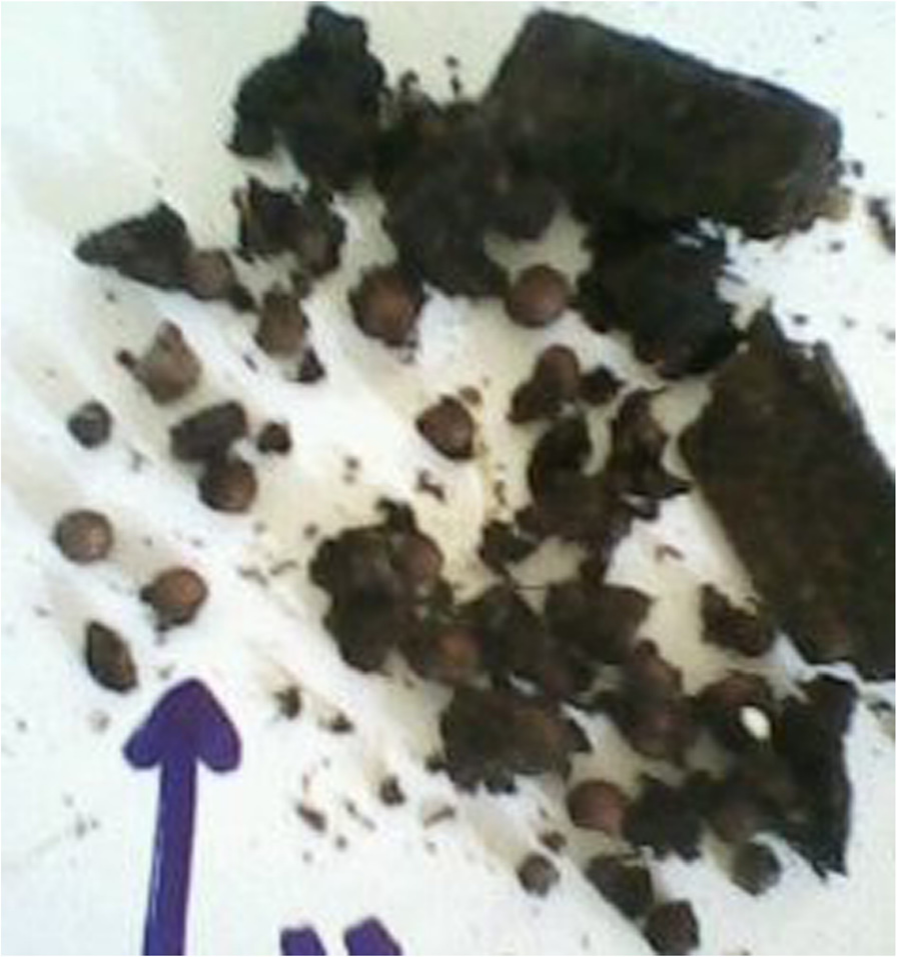
Boiled seeds from the pot

**Fig. 3:**
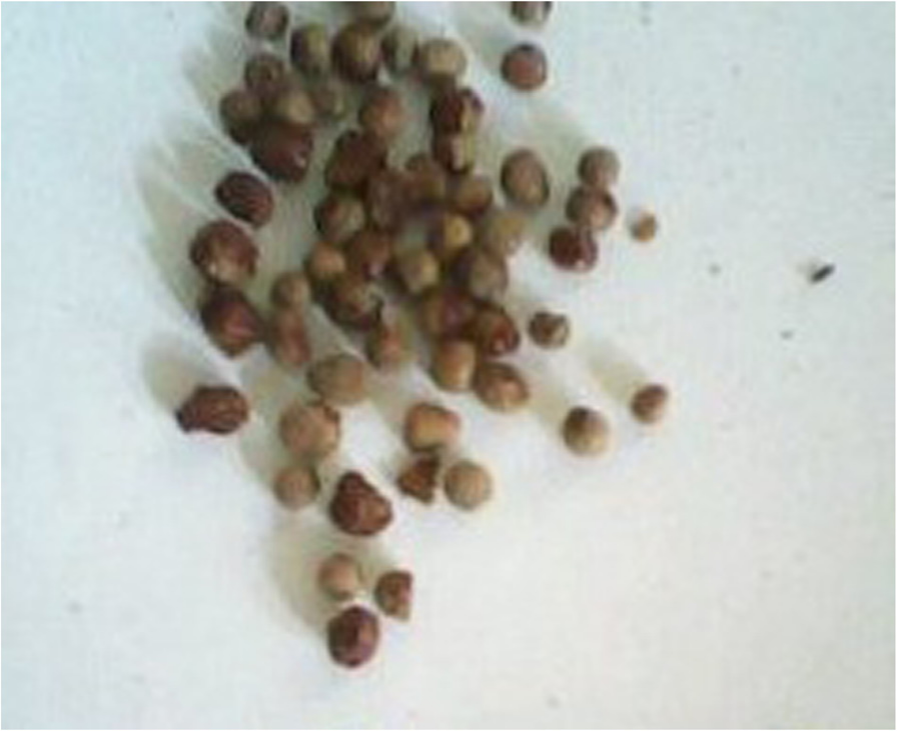
Gloriosa seeds from patients pocket

A 27-year-old man was admitted to the General Hospital Chilaw, Sri Lanka, with acute onset severe epigastric pain and vomiting. There was no history suggestive of food poisoning or the consumption of poison. A few hours before admission, the patient had consumed boiled coriander tea, which is a traditional medicine in Sri Lanka (Fig. 1). The tea was prepared for him by his sister-in-law as a treatment for common cold. The patient had developed symptoms about 2 h after drinking the coriander tea and he was rushed to the hospital. Other family members noticed that his sister-in-law was absent around the time of the admission and decided to examine the contents of the teapot. In addition to the coriander, they noticed that the pot also contained different seeds, which they correctly and promptly identified as *Gloriosa* seeds (Fig. 2). *Gloriosa* seeds were known to be present in the house because the patient worked on a farm that cultivated *Gloriosa*, and occasionally brought the seeds home (Fig. 3). Seeking confirmation, the wife of the victim tasted a small amount of the remaining tea and also developed nausea and vomiting. On admission to the hospital, the patient was having profuse watery diarrhea and complained of burning abdominal pain, reduced urine output, and dysuria. On examination, he was febrile with blood pressure of 100/70 mmHg and a heart rate of 100 bpm. The abdomen was diffusely tender and the respiratory rate was 20/min with oxygen saturation of 99 % on air. With prompt gastric lavage, the patient was started on intravenous ranitidine and metoclopramide and hydration with 0.9 % saline. On day two after poisoning, he complained of pleuritic-type chest pain and had tachypnea. His respiratory rate was 40/min with bilateral diffuse crepitation. Blood gas analysis revealed hypoxia with respiratory alkalosis and he was transferred to the intensive care unit (ICU) for intubation and ventilation. Chest X-rays showed mild diffuse inflammatory shadows, and blood counts revealed a white blood cell count of 17,000/mm^3^ with neutrophil leukocytosis of 90 % and lymphocytic leukocytosis of 8 % with a platelet count of 135,000/mm^3^. His CRP was 96 and ESR was 60 mm/1st hour.

On day three after admission, the patient had thrombocytopenia with a low platelet count of 60,000/mm^3^ and the white blood cell count was 7800/mm^3^ (N 55 %, L 40 %). In addition, he had mild renal impairment with serum creatinine of 1.4 mg/dl and blood urea of 60 mg/dl. There were also mildly elevated with AST of 60 U/L and ALT of 90 U/L with normal PT and APTT and bilirubin. ECG and 2D echocardiogram were normal. In addition to ventilator support, he was treated with broad-spectrum antibiotics (cefotaxime and metronidazole), parenteral nutrition, proton pump inhibitors, and intravenous fluids. The patient required ventilator support for 3 days and required high-dependency care for a further 6 days. His total hospital stay was 15 days. He also developed generalized alopecia by day 10 of hospital admission. By that time, the patient had fully recovered from acute toxicity and was hemodynamically stable.

Without the patient background and assistance from the patient’s family, this case could have been easily misdiagnosed at the acute stage and managed as a severe case of food poisoning, leptospirosis, or dengue shock with atypical presentation, which are relatively common presentations in the area. Colchicine poisoning would not have been suspected until the patient developed generalized alopecia toward the recovery around day ten of the illness [13]. Because there was reasonable suspicion of homicidal poisoning, the Judicial Medical Consultant and the hospital police post were informed to initiate medico legal investigation. The patient also admitted that he had taken *Gloriosa* seeds from his workplace where *Gloriosa* plants were grown for harvest and export of seeds. Toxicological analysis by the government analyst also confirmed the presence of colchicine in the gastric lavage samples.

## Conclusions

This patient showed classic complications of colchicine poisoning. He developed acute gastrointestinal symptoms and shock, followed by respiratory distress. He also developed mild thrombocytopenia and renal impairment. With his recovery progressed, he showed classical generalized alopecia. All of these complications reversed gradually with time and intensive medical care. Dried *Gloriosa* seeds look similar to coriander seeds and if mixed it would be difficult to differentiate for an unsuspecting person. *Gloriosa* grows as a wild plant in most parts of Sri Lanka, and it is extremely rare for someone to possess plant parts. This case is a rare example where the patient had access to seeds at his workplace. There are many plants that are poisonous and can be used as biological poisons in Asian countries [15]. In this case, *Gloriosa* seeds were used in an attempted homicide—an ideal poison to camouflage with coriander. Boiling the seeds in water would have extracted and concentrated the colchicine. Without the awareness of the family members of the availability of *Gloriosa* seed in this case, the intentional poisoning would have gone completely unsuspected in other circumstances.

## Abbreviations

ALT, alanine transaminase; APTT, activated partial thromboplastin time; AST, aspartate transaminase; CRP, C reactive protein; ECG, electrocardiogram; ESR, erythrocyte sedimentation rate; L %, lymphocytes percentage; N %, neutrophil percentage; PT, prothrombin time.
